# Human papillomavirus and p53 expression in cancer of unknown primary in the head and neck region in relation to clinical outcome

**DOI:** 10.1002/cam4.199

**Published:** 2014-02-10

**Authors:** Lars Sivars, Anders Näsman, Nikolaos Tertipis, Andrea Vlastos, Torbjörn Ramqvist, Tina Dalianis, Eva Munck-Wikland, Sushma Nordemar

**Affiliations:** 1 Department of Oncology-Pathology, Karolinska InstitutetStockholm, Sweden; 2 Department of Clinical Sciences, Innovation and Technology, Karolinska InstitutetStockholm, Sweden; 3 Department of Oto-Rhino-Laryngology, Head and Neck Surgery, Karolinska University HospitalStockholm, Sweden

**Keywords:** Cancer of unknown primary, head and neck cancer, human papillomavirus, p53

## Abstract

Patients with cancer of unknown primary (CUP) in the head neck region are generally treated with neck dissection followed by radiotherapy at times combined with chemotherapy, a treatment associated with considerable side effects. Some of these tumors may originate as human papillomavirus (HPV)-positive oropharyngeal squamous cell carcinoma (OSCC), with better clinical outcome than head neck squamous cell cancer (HNSCC) in general, and could potentially do well with less treatment. Here, we therefore investigated whether HPV status and p53-expression correlated to clinical outcome in patients with CUP in the head neck region. Fifty metastases were analyzed for presence of HPV DNA, and expression of p16^INK4A^ and p53 and the data were correlated to clinical outcome. Patients with HPV DNA-positive (HPV_DNA+_) metastases had significantly better 5-year overall survival (OS) compared to those with HPV_DNA−_ metastases (80.0% vs. 36.7%, respectively; *P *=* *0.004), with a similar tendency for disease-free survival (DFS). These survival rates showed excellent concordance with those of HPV_DNA+_ and HPV_DNA−_ OSCC in Sweden during the same time period, strengthening the hypothesis that HPV_DNA+_ head and neck CUP may originate from HPV_DNA+_ OSCC. In addition, having absent/intermediary-low as compared to high expression of p53 correlated to a better prognosis with a 69% as compared to 14% 5-year OS, respectively (*P *<* *0.001), and for DFS the tendency was analogous. In conclusion, both HPV status and p53 expression are valuable prognostic factors in patients with CUP in the head and neck region and should be further explored for clinical use.

## Introduction

Head and neck squamous cell carcinoma (HNSCC) is the sixth most common type of cancer in the world and may occur in the oropharynx, larynx, oral cavity, hypopharynx, the nasal cavity, and the sinuses 1. However, HNSCC can also present as a lump in the neck, which fine-needle aspiration cytology shows to be a lymph node squamous cell carcinoma metastasis. The patient undergoes a thorough diagnostic work up to find the primary tumor, including computed tomography (CT) or magnetic resonance imaging (MRI) of the head and neck region, CT of the chest, panendoscopy of the upper aerodigestive tract, tonsillectomy and blind biopsies from the base of tongue and epipharynx 2. This will reveal the primary tumor in most cases, and steer the treatment accordingly. However, in approximately 3–9% of all head and neck cancers, the primary tumor is never found, and these are denoted as “cancer of unknown primary” (CUP) in the head and neck region 3. These patients are treated with neck dissection in order to confirm the cytologic diagnosis with histopathology and postoperative radiotherapy, at times also chemotherapy 2, often leading to substantial side effects.

Traditional risk factors for HNSCC are tobacco and alcohol, but from 2007 human papillomavirus (HPV) has also been acknowledged as a risk factor for oropharyngeal squamous cell carcinoma (OSCC), a distinct subset of HNSCC 4. HPV-positive OSCC has a considerably better prognosis than HNSCC in general and OSCC are today, in many centers, treated primarily with oncological treatment and, if complete response is obtained, surgery can be avoided 5–11.

If the metastasis is HPV DNA-positive (HPV_DNA+_), it is likely that the patient has an HPV_DNA+_ OSCC as the primary tumor and the patient could be treated accordingly: for example, with radiotherapy or chemoradiotherapy and followed by neck dissection only in the case of noncomplete response. One aim of this study was therefore to examine for the presence of HPV DNA in lymph node metastases from CUP patients and investigate whether the presence of HPV DNA was correlated to 5-year overall survival (OS) and disease-free survival (DFS).

To detect HPV DNA, we used a bead-based multiplex assay. In addition, p16^INK4a^ and p53 were examined by immunohistochemistry (IHC), as p16^INK4a^ is often upregulated in high-risk HPV tumors, while p53 is mostly normal in HPV-positive tumors but mutated in smoking and/or alcohol-related cancer 12,13. Overexpression of p16^INK4a^ and p53 was analyzed in correlation to HPV status, smoking history and 5-year OS and DFS.

## Patients, Materials, and Methods

### Patients and material

One hundred patients whose primary initial diagnosis was CUP in the head and neck region (defined as ICD-10-code C.77.0) diagnosed between 2000 and 2007, at the Karolinska University Hospital, Stockholm, Sweden, were identified from the hospital database. Only patients treated with intention to cure and with available formalin-fixed paraffin-embedded (FFPE) material were included in the study. In total, 64/100 patients had available FFPE metastases. Of these 64 patients, six patients with histology other than SCC, two patients who only received palliative treatment, three patients who declined postoperative radiotherapy, and three patients with samples with insufficient tumor material for IHC, were excluded from the study. This resulted in that 50 patients with FFPE metastases included in the analysis. Patient data were collected from patient records and are presented in Table 1. All patients were treated with neck dissection and all patients, but one (see below) received postoperative radiotherapy (68 Gy). In four patients, a suspected primary tumor (three skin and one parapharyngeal tumor) were found, either during examination or at a later time, and one of these patients with a very small skin tumor, with the metastasis in the parotid gland did not receive postoperative radiotherapy. OS was defined as from the month of diagnosis until death by any cause. DFS was defined from the month of diagnosis until relapse. Patients who died without documented recurrence were censored at the date of death. Three patients who never became free of tumor were excluded from the calculation of DFS. Smoking was defined as ever or never (yes or no). The patients were followed up for a minimum of 60 months. Ethical permission was obtained from the Ethics board, Stockholm, Sweden, under Registration Number 2010/1758-31/4.

**Table 1:** Patient characteristics according to HPV DNA status in the metastasis.

Characteristic	All patients (*n *=* *50)	HPV_DNA+_ (*n *=* *20)	HPV_DNA−_ (*n *=* *30)	*P*
Gender, *n* (%)				
Male	37 (74)	16 (80)	21 (70)	0.522
Female	13 (26)	4 (20)	9 (30)	
Mean age (years)	65	62	67	0.130
N stage, *n* (%)				
1	14 (28)	7 (35)	7 (23)	0.655
2	31 (62)	11 (55)	20 (67)	
3	5 (10)	2 (10)	3 (10)	
Smoking history, *n* (%)^1^				
Yes	38 (78)	15 (79)	23 (77)	1.000
No	11 (22)	4 (21)	7 (23)	
p53 expression, *n* (%)				
0–60%	36 (72)	17 (85)	19 (63)	0.118
90–100%	14 (28)	3 (15)	11 (37)	

Figures in parenthesis denotes %. HPV, human papillomavirus; N, nodal.

1 Smoking data missing for one patient.

### HPV DNA analysis

DNA was extracted from 15-*μ*m slices of the FFPE metastases using the Roche High Pure RNA Paraffin Kit (Roche Diagnostics GmbH, Mannheim, Germany) according to the manufacturer's instructions. The *β*-globin gene was used in order to check that amplifiable cellular DNA was present in the samples and blank controls were included to exclude cross-contamination. HPV DNA detection was performed with a method developed by Schmitt et al. 14. HPV L1 and HPV16E6 were amplified from 10 ng sample DNA with broad-spectrum GP primers and HPV16E6 primers 15,16. HPV16 E6 was included to detect the rare cases where the HPV16L1 region was deleted in HPV-induced tumors. Amplicons for 27 HPV types, HPV16E6, and *β*-globin was assayed for in a bead-based multiplex assay on a Magpix instrument (Luminex Corporation, Austin, TX) as previously described with the result given as median fluorescence intensity (MFI) 14,16. Microsoft Excel was used for statistical analysis of the results. A MFI value above 1.5× background +15 was considered as a positive HPV signal. However, to exclude that tumors with a very low HPV content were regarded as HPV-positive, only samples with MFI values over 100 were considered HPV-positive in the further analysis. HPV-negative samples were excluded if the *β*-globin value was below 30 MFI as the DNA was considered of too poor quality.

### Immunohistochemistry

**Figure 1:**
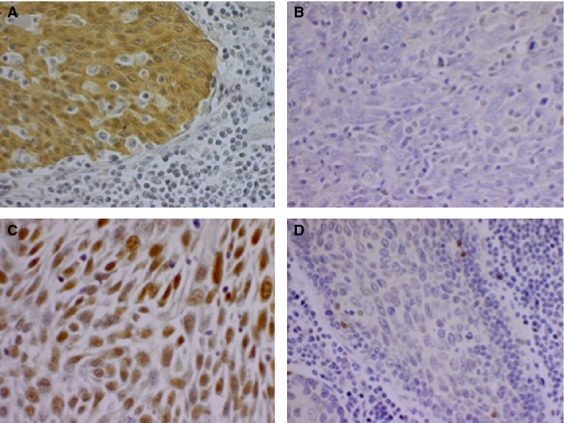
Representative photographs of p16 and p53 immunohistochemistry staining. A 40× magnification of (A) a p16-positive tumor sample; (B) a p16-negative tumor sample; (C) a sample with 100% of the tumor cells expressing p53; and (D) a sample with 0% of the tumor cells expressing p53.

In brief, tumor sections (4–5 *μ*m) were deparaffinized, rehydrated, rinsed in water, followed by antigen retrieval in citrate buffer (pH 6) for 20 min. Blocking of unspecific binding sites was done with 1% horse serum in phosphate-buffered saline (PBS) in a moist chamber for 30 min before the sections were stained with the primary antibody (mAb p53 (clone: DO-1, dilution 1:100, Santa Cruz Biotech, Santa Cruz, CA) that detects both mutated and wild-type p53 expression and mAb p16^INKA4a^ (clone: JC8, dilution 1:100, Santa Cruz Biotech) at +8°C overnight. The sections were thereafter washed in PBS and then incubated for 45 min at room temperature with the secondary biotinylated anti-mouse antibody (Vector Laboratories, Burlingame, CA), diluted 1:200 in PBS. The avidin–biotin–peroxidase complex (ABC) kit (Vectastain, Vector Laboratories) was used for antigen detection according to the manufacturer's instructions. Slides were developed in chromogen 3′-diaminobenzydine (DAB) (Vector Laboratories) with hematoxylin as a counter stain. All the IHC evaluations were conducted using light microscopy by an experienced researcher blinded for clinical data and outcome. p16^INK4a^ staining was evaluated as positive (with >70% positive tumor cells with strong intensity staining) or as negative as demonstrated in Figure 1A and B, respectively 8. The fraction of p53-positive cells was evaluated semiquantitatively in 11 grades of percentages of stained malignant cells: 0 (0%), 1 (1–9%), 2 (11–20%), 3 (21–30%) etc., and demonstrated for 100% and 0% p53 staining in Figure 1C and D, respectively. Absent/intermediate-low expression was defined as 0–60% of the cells being stained, while intermediate-high expression was defined as 61–89% of the cells being stained, and high expression with >90% of the cells being positive 17,18. Staining of tissue sections with both mouse monoclonal antibodies, and secondary antibody alone served as negative controls.

### Statistical analysis

Statistical calculations were performed using IBM SPSS Statistics software (Version 21.0; IBM Corp., Armonk, NY). Survival curves and 5-year OS and DFS were calculated using the Kaplan–Meier method and the log-rank test was used to compare the results. Hazard ratios (HRs) were calculated using multivariate Cox regression models. For calculating the significance of differences between categorical variables the chi-square test was used unless any of the variables was less than 5, then the Fisher's exact test was used instead. For differences concerning age, an independent *t*-test was used to calculate the significance between two groups. Two-sided *P*-values were reported for all analyses and *P*-values below 0.05 were considered as significant.

## Results

### Presence of HPV DNA and p16 expression in the metastases

All 50 metastases had amplifiable DNA as determined by amplification of the *β*-globin gene. HPV DNA was detected in 20 of 50 (40%) metastases in the bead-based multiplex HPV assay, with HPV 16 present in 19 samples and HPV 33 in the remaining sample. In addition, 21/50 (42%) samples exhibited p16 overexpression by IHC, and 18/21 (86%) p16 overexpressing samples were also HPV_DNA+_. The remaining three metastases that were positive for p16 IHC were HPV_DNA−_. The metastases from all four patients where a suspected primary tumor was found were HPV_DNA−_.

A comparison between patients with HPV_DNA+_ and HPV_DNA−_ metastases was carried out and is presented in Table 1. There were no major differences between the two groups, however, patients with HPV_DNA+_ metastases showed a tendency to be younger than those with HPV_DNA−_ metastases, but this difference did not reach significance.

### p53 expression in the metastases

In total, 24/50 (48%) of the samples showed no p53 IHC staining, while six (12%) showed <10%, six showed (12%) 10–60%, and 14 (28%) showed 90–100% p53-positive cells. There were no samples showing between 61% and 89% p53 staining. A comparison between patients displaying absent/intermediary-low (0–60%) and high (90–100%) p53 expression was then carried out (see Table 2). There was a correlation between smoking history and p53 overexpression (*P *=* *0.021), while all other characteristics failed to reach statistical significance, although a tendency was demonstrated between being HPV_DNA+_ in the metastases and absent/intermediary-low p53 expression (*P *=* *0.118).

**Table 2:** Patient characteristics according to HPV status and p53 expression.

Characteristic	0–60% p53-expression (*n *=* *36)	90–100% p53-expression (*n *=* *14)	*P*
Gender, *n* (%)			
Male	28 (78)	9 (64)	0.329
Female	8 (22)	5 (36)	
Mean age (years)	65	65	0.994
N stage, *n* (%)			
1	11 (31)	3 (21)	0.692
2	21 (58)	10 (71)	
3	4 (11)	1 (7)	
Smoking history, *n* (%)			
Yes	24 (69)	14 (100)	0.021
No	11 (31)	0 (0)	
HPV DNA			
Positive	17 (47)	3 (21)	0.118
Negative	19 (53)	11 (79)	
p16			
Positive	18 (50)	3 (21)	0.110
Negative	18 (50)	11 (79)	
HPV_DNA+_/p16+			
Positive	15 (42)	3 (21)	0.211
Other	21 (58)	11 (79)	

Comparison between patients with absent-intermediary/low (0–60%) and high (90–100%) p53 expression in their tumors (there were no patients with 61–89% p53 expression). Figures in parenthesis denotes %. HPV, human papillomavirus; N, nodal.

### HPV and p16 status in the metastases in correlation with clinical outcome

**Figure 2:**
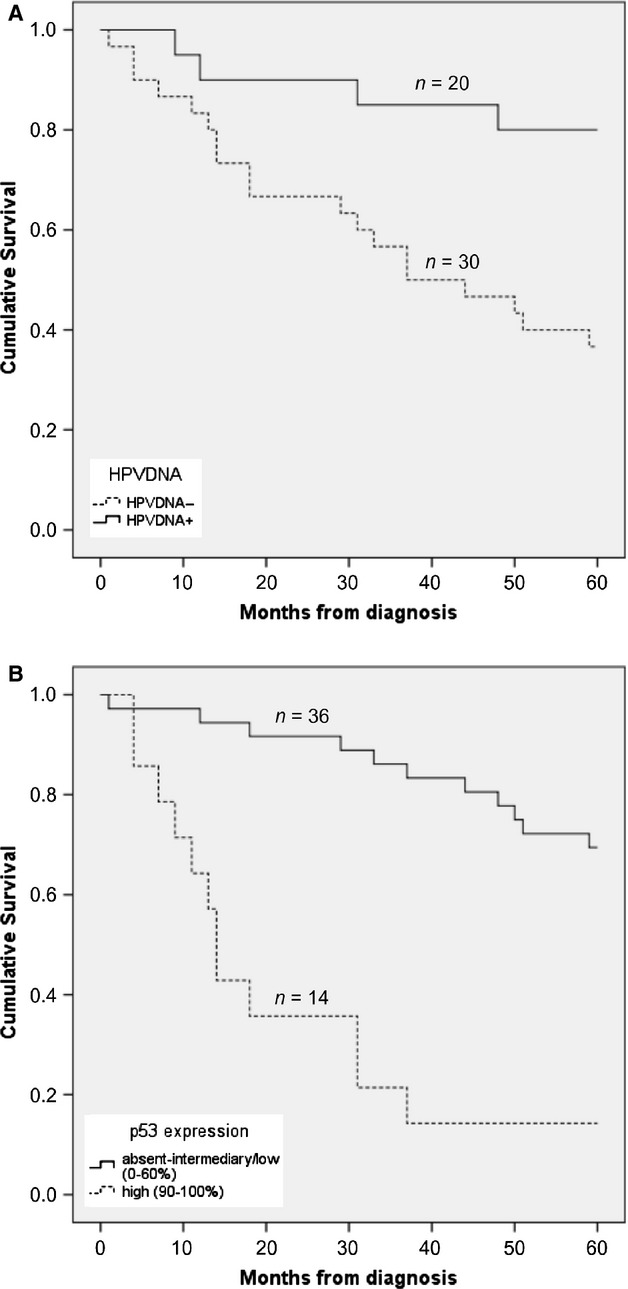
Effect of human papillomavirus (HPV) status and p53 expression on 5-year overall survival. (A) Cumulative survival in the HPV DNA-positive group and the HPV DNA-negative groups. (B) Cumulative survival in patients with absent-intermediary/low (0–60%) and high (90–100%) p53 expression. Notably, no patients showed 61–89% p53 expression.

The 5-year OS rate in the entire cohort, regardless of HPV DNA status, was 54.0%. The 5-year OS rate was significantly higher in the group with HPV_DNA+_ metastases (80.0%) compared to that in the HPV_DNA−_ group (36.7%) (*P *=* *0.004, log-rank test; Fig. 2A). Likewise, 5-year OS for the HPV_DNA+_ group was higher as compared to the HPV_DNA−_ group by univariate analysis, HR 0.236 (95% CI: 0.080–0.696, *P *=* *0.009; Table 3). A similar tendency was demonstrated when HPV-positivity was defined as being positive for both HPV DNA and p16 and compared to the patients with either HPV DNA or p16-negative metastases (77.8% 5-year OS vs. 40.6% 5-year OS, respectively, *P *=* *0.017, log-rank test). The two patients with HPV_DNA+_/p16-negative metastases both survived 5 years. The 5-year DFS was 85.0% in patients with HPV_DNA+_ metastases and 63.3% in patients with HPV_DNA−_ metastases. This difference, however, did not reach statistical significance (*P *=* *0.053, log-rank test).

**Table 3:** Presence of HPV DNA and p53 in the metastasis in relation to 5-year overall survival by uni-and multivariate analysis.

Characteristic	Univariate			Multivariate		
	HR	95% CI	*P*	HR	95% CI	*P*
HPV DNA status	0.236	0.080–0.696	0.009	0.290	0.092–0.913	0.034
P53 expression	6.561	2.789–15.436	<0.001	6.909	2.354–20.273	<0.001
Gender	0.935	0.368–2.372	0.887	2.763	0.911–8.386	0.073
Age	1.033	0.999–1.068	0.058	1.036	0.991–1.083	0.121
Smoking history	1.799	0.611–5.298	0.286	1.764	0.434–7.179	0.428

HPV, human papillomavirus; HR, hazard ratio.

When only p16 status was considered, 5-year OS was 76.2% in the p16-positive, as compared to 37.9% in the p16-negative group (*P *=* *0.007, log-rank test), while 5-year DFS was 85.7% in the p16-positive, as compared to 62.1% in the p16-negative group (*P *=* *0.032, log-rank test), data not shown.

### p53 expression in the metastases in correlation with clinical outcome

Expression of p53 was also significantly correlated to survival. The 5-year OS rate was 69.4% in patients with absent/intermediary-low (0–60%) p53 expression as compared to only 14.3% 5-year OS in the group with high p53 expression (≥90 staining) (*P *<* *0.001, log-rank test; Fig. 2B). Accordingly, the high p53-expressing group had a worse 5-year OS compared to the p53 absent/intermediary-low group by univariate analysis, HR 6.561 (95% CI: 2.789–15.436, *P *<* *0.001) (Table 3). The same tendency was shown when the 5-year DFS was calculated with 83.3% and 42.9% 5-year DFS in the absent/intermediary-low and high p53 expression groups, respectively (*P *<* *0.001, Log Rank test).

### HPV status and p53 expression in correlation with clinical outcome

**Figure 3:**
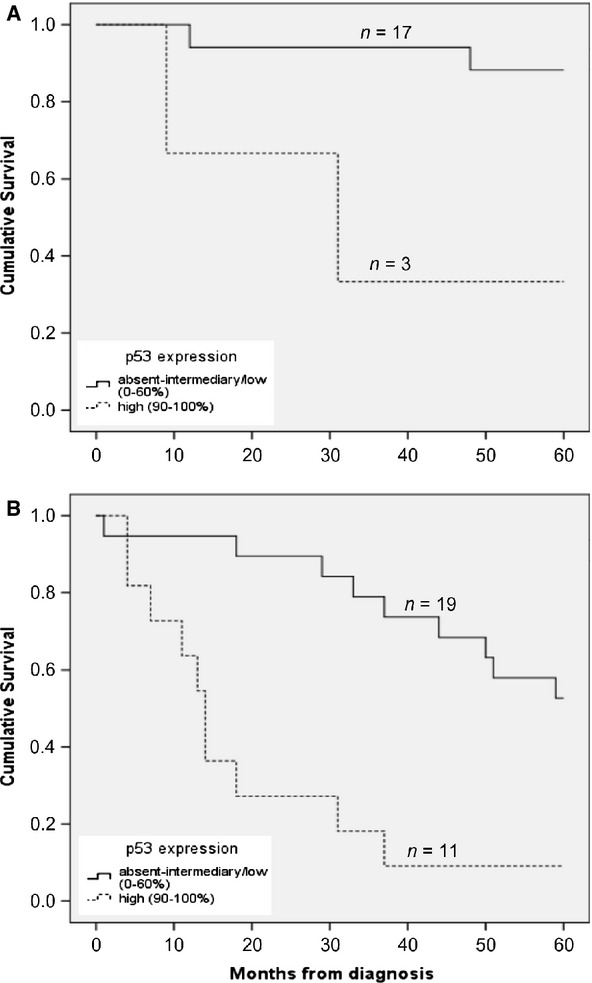
Effect of p53 expression on the survival in the human papillomavirus (HPV) DNA-positive group and in the HPV DNA-negative group. (A) Cumulative survival in patients with HPV DNA-positive metastases with absent-intermediary/low (0–60%) and high (90–100%) p53 expression. (B) Cumulative survival in patients with HPV DNA-negative metastases with absent-intermediary/low (0–60%) and high (90–100%) p53 expression.

In order to investigate whether p53 expression had an impact on survival independent of HPV DNA status, the 5-year OS survival rate was calculated separately for patients with HPV_DNA+_ and HPV_DNA−_ metastases and the data are presented in Figure 3A and B, respectively. Patients with HPV_DNA+_ metastases had a better 5-year OS when the tumors had absent/intermediary-low p53 expression as compared to high p53 expression (88.2% and 33.3% 5-year OS, respectively), and the data reached statistical significance (*P *=* *0.010, log-rank test; Fig. 3A). Patients with HPV_DNA−_ tumors and absent-intermediary/low p53 expression had a 52.6% 5-year OS as compared to a 9.1% 5-year OS in the high-p53 expression group (*P *<* *0.001, Log Rank test; Fig. 3B).

A multivariate analysis including HPV DNA, p53-expression, gender, age, and smoking habits (Table 3) showed that HPV_DNA+_ status conferred a survival benefit with a HR of 0.290 (95% CI: 0.092–0.913, *P *=* *0.034). According to the analysis, p53 overexpression was correlated with survival independently of HPV status with a HR of 6.909 (95% CI: 2.354–20.273, *P *<* *0.001) (Table 3).

### Survival according to smoking history, nodal spread, and age

Mean age correlated to the 5-year OS (*P *=* *0.036, independent two-tailed *T*-test). Patients without smoking history had a 5-year OS rate of 63.6% compared to 50.0% 5-year OS for smokers, this difference did not, however, reach significance (*P *=* *0.277, log-rank test). Patients with less-advanced nodal spread showed a.tendency toward better prognosis, but again, the data did not reach statistical significance (85.7% 5-year OS rate for N1 disease, 61.3% for N2 disease, and 60.0% for N3 disease (*P *=* *0.263, log-rank test). Gender did not correlate to the 5-year OS rate (*P *=* *0.886, respectively, log-rank test).

## Discussion

In this study, we have analyzed the influence of HPV status and p53 expression on clinical outcome in 50 CUP in the head and neck region. Forty percent of the CUP were HPV_DNA+_ and >70% of the CUP were either p53 negative or had >60% of the cells being positive for p53 by IHC. We found that both an HPV_DNA+_ status, as well as absent/intermediary-low p53 expression in the metastases independently correlated with a better clinical outcome as compared to that of patients with metastases, which were HPV_DNA−_ or had high p53 expression. More specifically, the 5-year OS rate was 80.0% in the HPV_DNA+_ group as compared to 36.7% in the HPV_DNA−_ group with a HR of 0.290. These survival rates showed excellent concordance with the survival rates for HPV_DNA+_ and HPV_DNA−_ OSCC in Stockholm, Sweden during the same time period 7,11. More specifically, these rates were 81% versus 36% 5-year disease-specific survival for HPV_DNA+_ and HPV_DNA−_ tonsillar cancer, respectively, and around 80% versus 37% 4-year OS for HPV_DNA+_ and HPV_DNA−_ base of tongue cancer, respectively 7,11. These data, thus further strengthen the hypothesis that HPV_DNA+_ head and neck CUP most likely originate from HPV_DNA+_ OSCC and consequently be treated as such 11.

In addition, p53 expression evaluated by IHC had an impact on survival. In patients with metastases with absent/intermediary-low p53 expression the 5-year OS was 69.4% as compared to only 14.3% 5-year OS in patients with high p53 expression (*P *<* *0.001). Similar survival benefits were demonstrated for 5-year DFS. Furthermore, a multivariate analysis showed that both p53 and HPV DNA status were independently correlated with survival. Moreover, we showed by combining HPV DNA status and p53 expression that the survival of patients with HPV_DNA+_ and p53 absent/intermediate-low CUP had an even better survival as compared to those with CUP that were only HPV_DNA+_ or had absent/intermediate-low p53 expression (88.2% vs. 80% vs. 69.4% 5-year OS, respectively). HPV DNA status and p53 expression could therefore both be considered prognostic factors in head and neck CUP and especially the combination of the two examined further in larger cohorts.

There are a few previous studies investigating the presence of HPV in metastases from head and neck CUPs in relation to prognosis. Compton et al. 19 investigated 25 patients in southern USA using HPV FISH and p16 IHC showing a similar trend as in this study with 67% 5-year OS and DFS in the HPV-positive group compared to 49% in the HPV-negative group. However, the difference in their study did not reach statistical significance (*P *=* *0.35 for OS; *P *=* *0.54 for DFS) likely, at least in part, due to the small study population. Furthermore, Tribius et al. 20 investigated 63 patients in a two-center study (Hamburg and Kiel) in Germany using p16 IHC and a PCR-based method for identification of HPV DNA. They found no differences in OS with a 76% 2-year OS in patients with HPV-positive metastases as compared to 75% in patients with HPV-negative metastases although a separation of the curves in their Kaplan–Meier plots could be noted over time 20. The result in this study, with 40% of the patients positive for HPV DNA and 36% of the patients positive for both HPV DNA and p16 IHC, is in line with the results of Compton et al. and Tribius et al. (28% and 37%, respectively, positive for both HPV DNA and p16 IHC) 19,20. Based on the findings of Crompton et al., Tribius et al., and us, where we all find a significant number of patients with HPV_DNA+_ p16^INKA4a^-positive CUP that have a tendency to have a better clinical outcome compared to those with HPV_DNA−_ p16^INKA4a^-negative CUP, further studies are warranted in this patient group 19,20. It would be extremely useful, if one could analyze for presence of HPV already in the fine-needle aspiration cytology (FNAC) and compare clinical outcome in patients according to HPV status in the FNAC. If the clinical outcome of patients with HPV-positive FNAC turns out to be better than that of patients with HPV-negative FNAC, then prospective randomized clinical trials could be initiated avoiding neck dissection.

There are to our knowledge no previous studies analyzing p53 expression as a prognostic marker in head and neck CUP. There are, however, numerous studies on p53 in general HNSCC, but a systematic review shows that the evidence of the prognostic value of p53 is inconclusive and that more research is needed 21. It also appears as if there are differences between different subsets of HNSCC whether a *high* expression of p53 or a *low* expression of p53 confers a survival benefit 21. In OSCC, some studies show that a high degree of p53 expression is a marker for a worse prognosis, while others find no difference in survival between high and low p53 expression 22,23. HPV status and p53 expression is often linked, with p53 degradation seen in cells infected with high-risk HPV 24. It is therefore not surprising that we show how a low p53 expression confers a survival advantage, as HPV-positive OSCC has a better prognosis than HPV-negative OSCC 5–11. However, the results of the present study indicate that p53 has a role as a prognostic factor independent of HPV status. The mechanism behind this observation is not known and further more extensive studies on the role and impact of p53 would be of interest. Nevertheless, the combination of HPV status and p53-expression tended to give additional prognostic value for head and neck CUP patients. Of note, the two HPV_DNA+_/p16 CUP both had absent p53 expression.

Although we demonstrated a trend toward a better prognosis for nonsmokers compared to smokers (63.6% vs. 50% 5-year OS survival, respectively), the data did not reach statistical significance (*P *=* *0.277), although smoking is a well-established negative prognostic factor in HNSCC including OSCC 4. It is also interesting that we did not find a significant difference in smoking history between patients in the HPV_DNA+_ and HPV_DNA−_ groups, as we expected, since this is often seen in OSCC 7,8. One possible explanation of this is our relatively small study population; another is suboptimal smoking data from patient records. It has been shown that smoking decreases survival in OSCC in proportion to the number of pack-years smoked and it is common to use 10 pack-years as a cutoff for defining a patient as a smoker or nonsmoker in HPV-related cancer studies 8. Due to the retrospective character of this study and the incomplete data concerning smoking habits in the patient records, smoking was defined as ever or never (yes or no) instead of the more desirable number of pack-years. It is plausible that we would have obtained significant results if there had been better data on pack-years.

The mean age was lower in the HPV_DNA+_ group as compared to the HPV_DNA+_ group (62 vs. 67 years), although this difference failed to reach significance (*P *=* *0.130), showing a similar trend as in OSCC, where patients with HPV-positive OSCC usually are younger than those with HPV-negative OSCC 9,16. The mean age in our cohort was 65 years, which was higher than that in the studies of Tribius and Compton (58 and 60 years, respectively) 19,20. In line with the proportions seen in OSCC and HNSCC in general, and in the studies of Tribius et al. and Comptons et al., 74% of the patients in our cohort were males 19,20,25,26. Nodal status is an established prognostic factor in CUP 26. We, however, did not find a statistically significant difference in survival (*P *=* *0.263), most likely due to our small study population. Nevertheless, a trend toward better outcome was seen in patients with less advanced nodal spread.

The study limitations to be considered are first, that this was a retrospective cohort study and the results need to be confirmed prospectively. Second, the study population was small. This is, however, difficult to avoid when dealing with such a rare entity as head and neck CUP and it is worth pointing out that we reached statistical significance for several parameters. Nevertheless, our data suggest that HPV status and p53 expression in lymph node metastases are potentially interesting as prognostic markers in head and neck CUP.

In conclusion, in patients diagnosed with head and neck CUP, the independent presence of HPV-positive status, or absent/intermediary-low p53 expression in the lymph node metastases, correlated to a better 5-year OS for the patients as compared to those with metastases with HPV-negative status or high p53 expression. This suggests that it would be worthwhile to confirm these studies in a larger group of patients and that the combined analysis of both HPV status and p53 expression may be useful and preferable for individualizing treatment for CUP patients in the future. In the case of a reliable cytological squamous cell carcinoma diagnosis, the finding of HPV-positive cancer cells may be sufficient to consider the primary tumor to be an undetectable OSCC and treat the patient accordingly.
